# Mechanistic understanding of nanoparticles’ interactions with extracellular matrix: the cell and immune system

**DOI:** 10.1186/s12989-017-0199-z

**Published:** 2017-06-24

**Authors:** Ayse Basak Engin, Dragana Nikitovic, Monica Neagu, Petra Henrich-Noack, Anca Oana Docea, Mikhail I. Shtilman, Kirill Golokhvast, Aristidis M. Tsatsakis

**Affiliations:** 1 Department of Toxicology, Faculty of Pharmacy, Gazi University, Hipodrom, 06330 Ankara, Turkey; 2 Laboratory of Anatomy-Histology-Embryology, Medical School, University of Crete, Heraklion, Greece; 3 “Victor Babes” National Institute of Pathology, Immunology Department, 99-101 Splaiul Independentei, 050096 Bucharest, Romania; 4 Institute of Medical Psychology, Otto-von-Guericke University, 39120 Magdeburg, Germany; 5 Department of Toxicology, University of Medicine and Pharmacy, Faculty of Pharmacy, Petru Rares, 200349 Craiova, Romania; 6 Master School Biomaterials, D.I. Mendeleyev University of Chemical Technology, Moscow, Russia; 7 Scientific Educational Center Nanotechnology, Engineering School, Far Eastern Federal University, Vladivostok, Russian Federation; 8 Center of Toxicology Science & Research, Medical School, University of Crete, Heraklion, Crete Greece

**Keywords:** Extracellular matrix, Nanoparticle, Inflammation, Biological barriers

## Abstract

Extracellular matrix (ECM) is an extraordinarily complex and unique meshwork composed of structural proteins and glycosaminoglycans. The ECM provides essential physical scaffolding for the cellular constituents, as well as contributes to crucial biochemical signaling. Importantly, ECM is an indispensable part of all biological barriers and substantially modulates the interchange of the nanotechnology products through these barriers. The interactions of the ECM with nanoparticles (NPs) depend on the morphological characteristics of intercellular matrix and on the physical characteristics of the NPs and may be either deleterious or beneficial. Importantly, an altered expression of ECM molecules ultimately affects all biological processes including inflammation. This review critically discusses the specific behavior of NPs that are within the ECM domain, and passing through the biological barriers. Furthermore, regenerative and toxicological aspects of nanomaterials are debated in terms of the immune cells-NPs interactions.

## Background

**Fig. 1:**
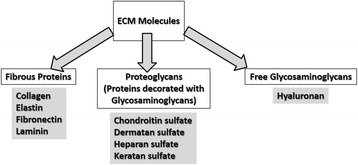
Classification of ECM molecules

Extracellular matrix (ECM) represents a complex network of variously modified proteins and the glycosaminoglycan, hyaluronan, highly organized in a form of a suprastructure which ultimately constitutes the cell microenvironment [1]. The ECM proteins are usually classified into two main categories: fibrous proteins constituted of various collagens and elastin, and glycoproteins including fibronectin, glycosaminoglycan decorated protein-proteoglycans and laminin [2] (Fig. 1). Cells are embedded in hydrogels comprising these different biopolymers and proteoglycans that fill the extracellular space [3]. Moreover, the ECM architecture is highly specialized and tightly regulated as the result of inherent properties of the component molecules, as well as of the biologic potency of the dwelling cells. The ECM is a fundamental component of the microenvironment of cells exhibiting both cell and tissue type specificity, which facilitates cell biological functions and defines tissue properties. Concisely, the ECM components provide the mechanical and structural support as they define the size, morphology and strength of tissues *in vivo* [4]. Additionally, this polymer-based microenvironment is also important during growth, development, and wound repair as well as key to various disease processes. Therefore, it acts as a pool for signaling molecules including inflammatory mediators, transporting signals from other origins to proliferating, differentiating and migrating cells [5–7].

The evolution of nanotechnology and application of nanoparticle (NP) based materials in medicine have opened a new era in diagnosis and treatment improvement for several health issues. Considering the scanty amount of information about the inflammatory changes of the ECM interacting with the NPs, this review aims to elucidate the interplay and accumulation of NPs by the ECMs. It will outline key chemical, physical, and biological properties that influence distinctive domains of the neighboring biological barriers and the resultant effects on homeostasis and immune processes.

## Extracellular matrix

### Basic components of Extracellular matrix and role of inflammation

Historically, the ECM components were defined as inert “mucilage” which surrounds the cells and were thereby placed on the secondary-track of innovative and pioneering research endeavors. During the last decades, however, breakthrough advances in the field have been achieved and these biomolecules are presently acknowledged as indispensable participants in all vital biological processes and nanomedicine applications [1]. Indeed, it is now well established that the biomolecules that constitute the ECM have developed structural and physico-chemical properties that particularly meet the requirements necessary for the execution of their specific biological functions in their individual tissues. Thus, these macromolecules are composed of independent structural domains which in turn form homopolymers and heteropolymers that become supramolecular assemblies with highly specialized structure and functions [2, 8]. The domain organization of ECM molecules may be correlated to conserved structure–function relationships, including the binding of specific integrin or non-integrin receptors by fibronectin, fibrillation of collagen or its decoration by mediator-binding small leucine rich proteoglycans thereby affecting the topical concentration or availableness of these factors [4, 9]. These assemblies thus, contain binding domains for different cytokines as well as ligands for canonical growth factor receptors, form complex adhesion/interacting surfaces and create diffusion boundaries between neighboring biological layers [8, 10]. In summary, each class of ECM molecules has developed unique physical and signaling properties which enable interaction with other ECM components to ultimately facilitate correct tissue organization, growth and function [2].

**Fig. 2:**
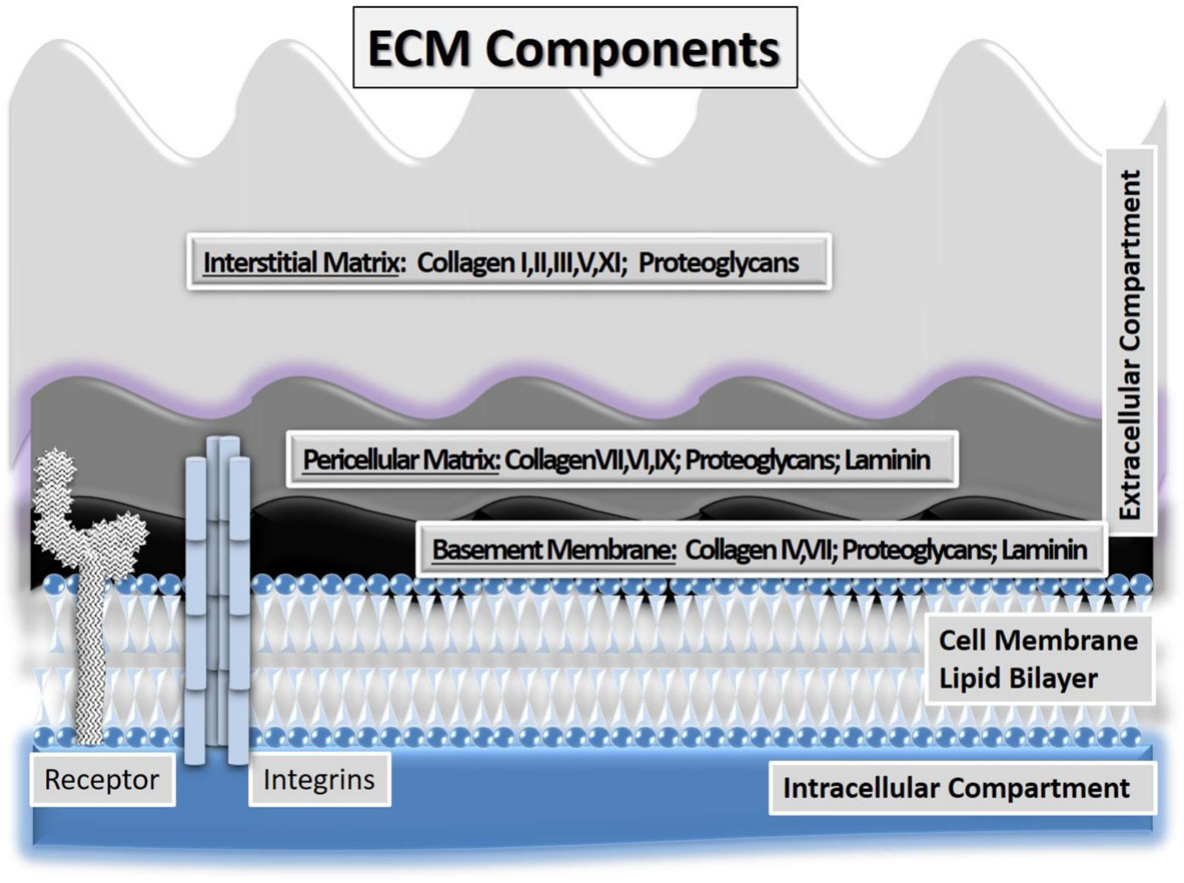
Components of ECM

The two basic structures of the ECM are basement membranes, which are organized as thin layers of highly crosslinked biomolecules, and the loose array of fibril-like macromolecules which in fact form the interstitial matrix. Specifically, the interstitial matrix is a complex composed of the collagens organized as fibrils (such as collagen types I, II, III, V and XI), which provide plasticity and tensile strength and of non-collagenous glycoproteins (such as tenascin, fibronectin, chondroitin/dermatan sulfate containing proteoglycans), which are highly negatively charged molecules with an outstanding capacity for intermolecular interactions with other ECM components and cytokines [11] (Fig. 2). Pericellular matrices, are a very narrow zone found in the immediate vicinity of cells, with specific characteristics. This region has been shown to be rich in proteoglycans (e.g., aggrecan and decorin), collagen (types II, VI, and IX), fibronectin and hyaluronan but is defined primarily by the presence of type VI collagen [12].

**Fig. 3:**
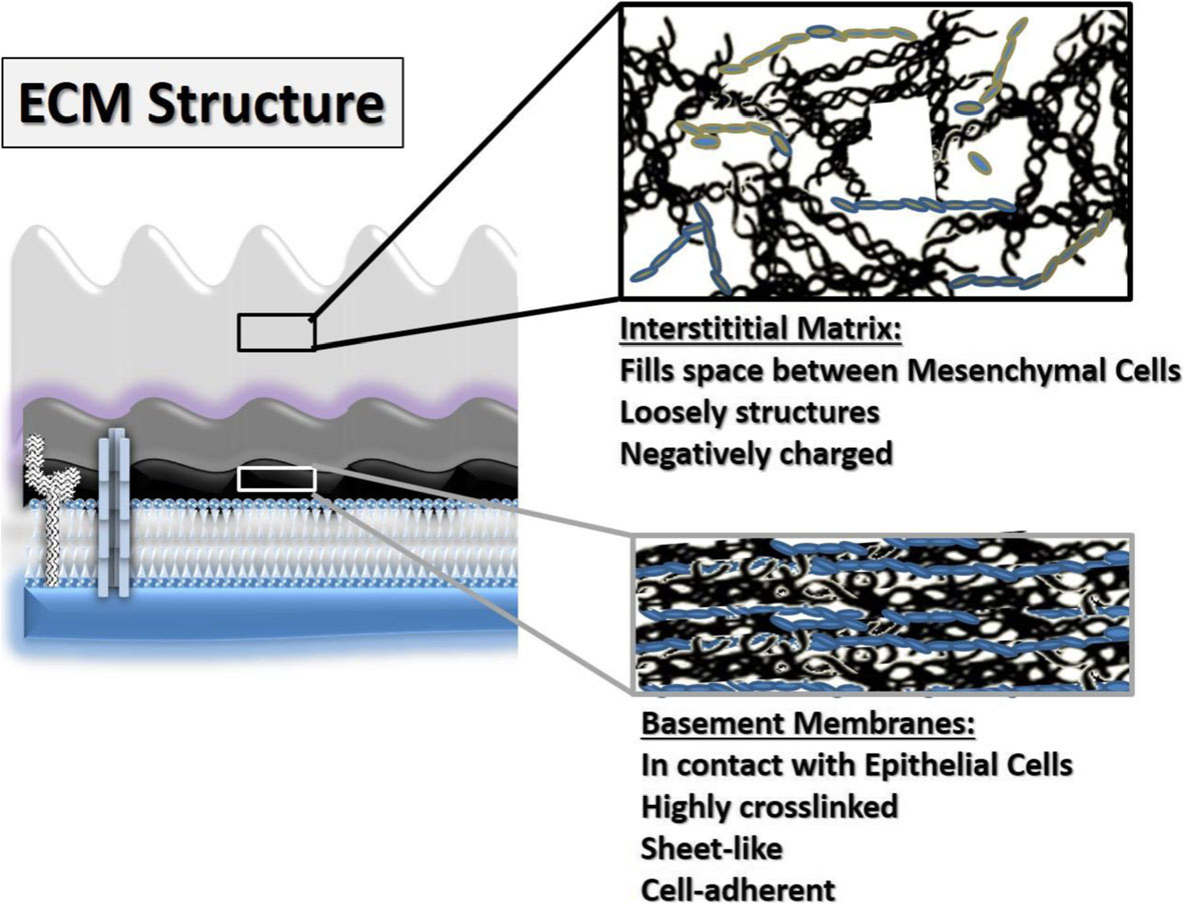
Schematic presentation of localization of basic ECM structures

The basal membranes are organized layers of cell-adherent ECM molecules that form supporting platforms for “anchored cells” including epithelial, muscle, nerve as well as endothelial cells [13, 14] (Fig. 3). They are mostly formed of collagen IV and VII crosslinked polymers, heparan sulfate containing proteoglycans as well as laminins bound to basement membrane protein, nidogen [15]. These “sheet-like” structures are formed early during embryogenesis in order to segregate developing tissues, function as macromolecular filters and provide sites for cell adhesion [14]. Importantly, the basal membranes shield tissues from deleterious biological stresses while concurrently facilitating dissemination of information among cells residing in the tissue and between cells and their exterior microenvironment [13, 14]. Furthermore, highly specialized ECM structures with features common to the basement membrane and the interstitial matrix, create the reticular fiber web of secondary lymphoid organs [11].

The ECMs are essential for tissue homeostasis while their remodeling is closely correlated to different pathological conditions including inflammation, cancer and toxicity related to xenobiotics [6, 16, 17]. A significant number of reports testify to an important role of ECM in inflammatory processes [18–20]. Indeed, it is widely accepted that ECM regulates various features of the inflammation process including the extravasation of leukocytes and their subsequent migration through the interstitial space [18]. Then again, in inflamed tissues, both the turnover of ECM components and the secretion of proteases by cells residing in tissues are regulated by cytokines that are secreted by the activated infiltrating cells [6]. There are strong indications in favor of the fact that ECM breakdown products are not only the consequence of inflammation, but also key players in the protraction of the inflammatory process. Changed expression of ECM molecules can in turn affect the activation, differentiation and survival of immune cells, and thus ultimately influence the extension of the inflammatory response and the evolvement of a chronic course [6]. Understanding the interaction of ECM and inflammatory processes is pivotal for medicine as most diseases are associated with secondary inflammatory mechanisms (e.g. ischaemia or metabolic diseases).

### Extracellular matrix and nanoparticles interactions

It is now increasingly recognized that NPs interact with the complex suprastructure of the ECM [21, 22]. Moreover, the territorial, immediately surrounding cell region of the ECM, the pericellular matrix, may have an important contribution in the interplay among NPs and various biological barriers. NPs are materials with novel physico-chemical properties classified due to their size, structure and surface chemical modifications. Indeed, the European Union published a definition on nanomaterials (2011/696/EU) which was adopted by the European Commission in October 2011. “The published Recommendation on the Definition on Nanomaterials defines nanomaterial as a natural, incidental or manufactured material containing particles, in an unbound state or as an aggregate or as an agglomerate and where, for 50% or more of the particles in the number size distribution, one or more external dimensions is in the size range 1 nm-100 nm (2011/696/EU).” [23]. NPs are influenced by components of the ECM in three ways: (i) they collide with matrix fibers as they diffuse near fibers, (ii) restricted thermal motion of water molecules due to proximity to the fibers slows NPs’ diffusion (hydrodynamic interactions), and (iii) for charged particles, electrostatic interactions with charged components of the ECM present an additional, limiting force [24].

NPs interact with the biological system, often leading to the induction of inflammatory or allergic reactions, as well as to activation of the complement system [25] on one hand. On the other hand, NPs play the role of carrier and adjuvant, with the immune response being dependent on size and crystallinity [26]. Transport of the NPs through the ECM is more complicated due to its’ inherent mesh-like organization [27]. The combined density of all participating biopolymers sets the physical properties of matrix. Particles with diameters larger than the size of network space are rejected by ECM, while smaller particles are able to pass through the matrix barrier [3]. Furthermore, it is well-known that changes in the microenvironment and the ECM surrounding the cells can profoundly influence signal transduction events into and out of the cells [28]. The 20–40 nm spacing between collagen fibrils block the migration of larger particles. Additionally, inter-fibrillar spacing, 75–130 nm around the compact collagen bundles or within loose bundles hampers the diffusion sets another limit for particle size [29]. Noteworthy, the alignment and spacing of collagen fibrils is arranged by proteoglycans. The space among the collagen fibrils is determined by dermatan/chondroitin sulfate or keratan sulfate chains bound into the protein cores of small leucine rich proteoglycans. The proteoglycan protein cores bind to the specific binding domains of collagen fibrils at approximately every 65 nm while their sulfated side chains bridge and determine the inter-fibrillar space [30]. Likewise, the basement membrane hinders the diffusion of NPs in a manner dependent on their size. Indeed, the specialized network of ECM proteins substantially affect the transmucosal transport of NPs [31]. Thus, it is important to note that the ECM components -NPs interactions are dependent on their respective intrinsic properties [32]. As regarding the ECM components, although the size of glycosaminoglycan chains is a few nanometers, their effect is important. Thus, negatively charged chondroitin sulfate content of the ECM, restricts uptake of NPs. The charge of NP as well as the charge of the ECM components are critical for trafficking and uptake of NPs [33]. In contrast, for larger fibers such as collagen, the effect of repulsive forces becomes less significant. Also, when comparing the parallel and transverse to the preferred fiber direction diffusion coefficients to the preferred fiber direction it was demonstrated that the mobility of charged particles is affected more in the transverse direction [24]. Therefore, the complex ECM hydrogel can be interpreted as a network with localized charge patches. These patches in the ECM are responsible for its highly unspecific but strongly selective filtering effect for NPs [3]. Regarding the modulation of other biological functions, it can be noted that molecular spacing is also important for cell adhesion [34]. Cells adhere well to nanopatterned agrin, which is a heparan sulfate proteoglycan, when presented as uniformly coated substrate. However, there is a threshold for the distance between the NPs to facilitate the adhesion. The adhesion dramatically decreases when the space between agrin-coated NPs broaden from 60 to 90 nm. This shows that cell adhesion to nanopatterned substrates primarily corresponds to the interactions with the ECM rather than to the transmembrane adhesion [35]. All cells with exception of blood cells have the ability to adhere onto the components of the surrounding ECM [4]. In summary, one aspect of the interaction of the ECM and NPs is the filtering effect of the relatively densely structured ECM with its distinct charge properties. Thus, very small NPs with a size less than 20 nm may not be hindered and may therefore exhibit a higher toxicity risk. Thereby, the data discussed likely suggests that NP effects on cells can be modulated in a location specific manner by the constituents of the surrounding ECM.

### Toxicity of nanoparticles to cells and ECM components

The size of NPs determines their toxicity to a large extent. Indeed, an inverse relation between particles smaller than 100 nm and their toxicity has been established with the NPs inducing a time- and dose-dependent decrease in cell viability. Moreover, the agglomeration of NPs results in altered mechanical properties [32]. In addition to size, the shape and charge of the NPs contribute crucially to the behavioral characteristics of particles in the biological systems [36–38]. In this respect, carbonate form layered double hydroxide NPs that are anionic lamellar compounds made up of positively charged brucite-like layers, exhibit higher toxicity compared with the chloride form in terms of induction of oxidative stress, apoptosis and membrane damage [39, 40]. The chemical surface structure of the amorphous silica NP (aSNP) defines the toxicological outcomes of the particle. Upon aspiration, the aSNP attaches to the negatively charged alveolar surfactant layer with a thickness of 10 to 20 nm. The ECM protein microfibril-associated protein (MFAP4) binds the collagen-like region of surfactant protein-D (SP-D). MFAP4 may fix the Sp-D in the extracellular compartment during NP induced inflammation [41]. Moreover, aSNP-NH_2_ causes a moderate toxic effect, while the carboxylated form does not appear to be cytotoxic. However, a significantly augmented interleukin-8 (IL-8) release is observed in the latter case, indicative of immune activation [42]. The uptake and interaction of the NPs with the cells are, however, not only affected by their surface charge, but also by respective ligands. Thus, the coupling of ligands on the surface of NPs can facilitate the cellular binding [43]. The number of coated human serum albumin molecules per NP is likewise influenced by their surface charge. Thereby, positively charged NPs are incorporated by cells to a larger extent than negatively charged ones, both in serum-free and serum-containing media [44]. On the other hand, hyaluronan a highly negatively charged glycosaminoglycan component of the ECM strongly attracts the citrate-gold NPs [45, 46]. Electrostatically stabilized citrate-coated very small superparamagnetic iron oxide particles (VSOP) bind to the cell surface with high affinity. However, inhibition of glycosaminoglycan synthesis by glucose deprivation in THP-1-derived macrophages is associated with a significant reduction of VSOP linkage. This effect is due to the high affinity of VSOP for the negatively charged glycosaminoglycans which are localized to the cell surface [47]. In addition, NPs can be modified with amphiphilic polymers which results in obtaining NPs of different charge but, with otherwise identical physical properties. While the protein-cationic NPs complex binds to cell surface scavenger receptors, the protein-anionic NPs complex binds to native protein receptors [48].

**Fig. 4:**
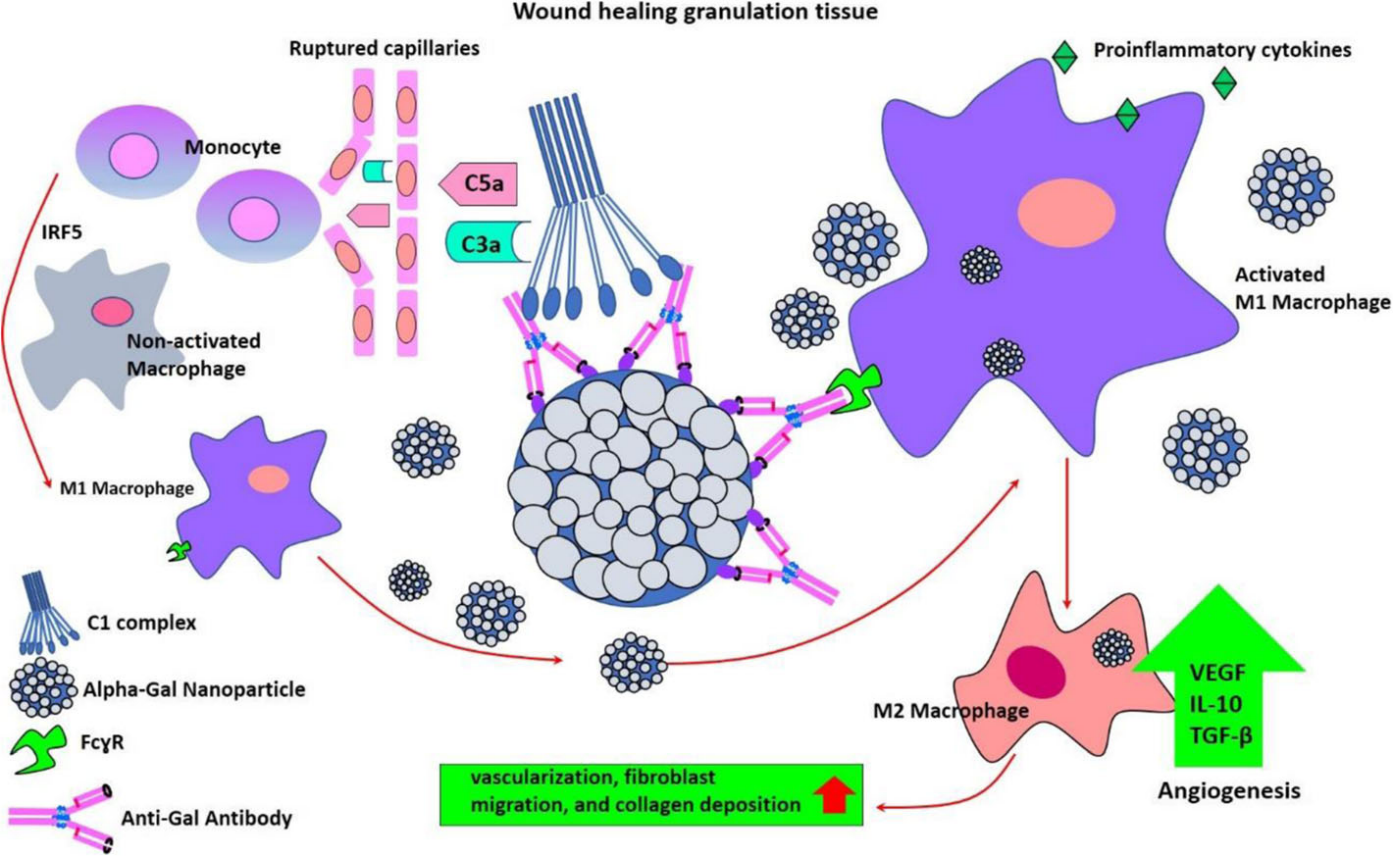
Anti-Gal antibody/ alpha-gal liposome interaction promotes wound healing by recruiting and activating macrophages

AgNPs composite nanofibers accelerate the rate of collagen production in comparison to plain collagen nanofibers, during wound repair [49]. Likewise N-acetylcysteine S-nitrosothiol NPs attenuate wound expansion and accelerate wound healing by increasing collagen deposition, as well as increasing M2 macrophage and decreasing neutrophil infiltration to the wound [50]. While the M1-polarized macrophages are involved in pro-inflammatory response, M2 macrophages exhibit an anti-inflammatory property during wound-healing. Furthermore, NP-mediated delivery of interferon regulatory factor 5 (IRF5) siRNA results in the resolution of inflammation by decreasing the recruitment of M1 macrophages and promoting the polarization of macrophages into M2 type in the wound [51]. NO-releasing NPs modify leukocyte migration and stimulate transforming growth factor-β (TGF-β) production, resulting in the subsequent promotion of angiogenesis, as well as the migration of fibroblasts and collagen deposition in wounded area resulting in accelerated wound healing [52] (Fig. 4).

In general, the risk of toxic effects of NPs increases with decreasing size, positive charge and fiber-like shape. In addition, surface ligands may be important for activation of the immune system. Therefore, it is of outmost importance to correctly correlate the fine modulations of NPs inherent characteristics with their biological effects.

### Nanoparticles and cell membrane interaction

The plasma membrane of the cells contains microdomains that are enriched in certain glycosphingolipids, gangliosides, and cholesterol that form membrane/lipid rafts. Membrane/lipid rafts have myriad functions including the regulation of cellular polarity and organization of trafficking and sorting mechanisms. These rafts are also important for forming platforms for ECM adhesion and intracellular cytoskeletal binding to the plasma membrane and for adherence to the ECM. Furthermore, they are involved in generation of signaling events and constitute the sites where NPs enter the cells [53].

Sphingolipid metabolites, ceramide, sphingosine, and sphingosine 1-phosphate (S1P) are known to act not only as intracellular second messengers, but also serve as important mediators in the extracellular space with the S1P binding to the cell surface S1P receptors [54]. Particle properties triggering intracellular reactive oxygen species (ROS) may be considered as determining factors for the subsequent adverse signaling. Pure carbon NPs are known to generate ROS due to their high surface area and the specific surface reactivity [55]. Oxidative stress can lead to cell dysfunction or cell death and can induce damage to the ECM [56]. Generation of intracellular oxidative stress as the initial event in a cascade of membrane signaling events involving the accumulation of sphingolipid metabolites in lipid rafts and the activation of the membrane-coupled epidermal growth factor receptor (EGFR) [57]. The most well-known localization of EGFR is to lipid rafts, which are enriched in cholesterol, sphingolipids and gangliosides [58]. Autophosphorylation of EGFR leads to the activation of two downstream pathways: The mitogen-activated protein kinase (MAPK) pathway and the phosphatidylinositol 3-kinase/ protein kinase B (PI3K/Akt) pathway. The major MAPK pathways consist of extracellular signal-regulated kinase (ERK)1/2, p38 and c-Jun N-terminal kinase (JNK) [59]. On the other hand, plasma low density lipoproteins (LDLs) complexes can be defined as native 20- to 25-nm NPs, which bind to the ECM proteoglycans in the arterial intima induce hydrolytic and oxidative modifications [60]. The interaction of LDL and lipoprotein lipase with four atheroma constituents, namely, smooth muscle cells, ECM, lipoprotein lipase and sphingomyelinase, represents a physiological process for solid, focal retention and aggregation of atherogenic lipoproteins in the arterial wall accompanied with macrophage foam cell formation [61].

NP-cell membrane interactions may affect the localization of the NPs, their intracellular trafficking, compartmentalization into various parts of the organism, and cellular retention [62]. Poly(dl-lactide co-glycolide, PLGA) NPs functionalized with poly-l-lysine adhere to the human breast carcinoma cell membrane with five-fold greater affinity and are rapidly internalized compared with the unmodified NPs [63]. In addition, the density of the NPs’ cationic charge, lipid bilayer’s negative charge load, surface tension, temperature, and salt concentration influences the capacity of particle to form a membrane “hole”. Cationic particles cause the formation of disintegrated surface unity in the bilayer and hydrophilic pores, allowing thus, the cationic NPs more readily penetrate through cellular membranes compared to the anionic counterparts [64]. Cationic lipid/DNA complexes (lipoplexes) are engulfed into cells by endocytosis or direct penetration through the cell membrane, following the association of the cationic lipopolyamines with membrane proteoglycans. Endosome–lysosome transition is delayed *via* the acidification of endosomes that is slowed down by the protonable amine groups of lipoplexes [65]. On the molecular level, the membrane proteins can be rearranged laterally. The conformational changes regulate the activity of these proteins. In addition, mechanical forces modulate the properties and the binding capabilities of receptor-ligands [66]. Thereby, NPs can also alter the plasma membrane organization by interaction with membrane-bound proteins. Thus, binding of the NPs to reduced nicotinamide adenine dinucleotide phosphate (NADPH)-oxidase (NOX) leads to generation of ROS and intracellular Ca^2+^ changes. Calcium promotes activation of NP-treated macrophages leading to the release of intermediates that activate intercellular adhesion molecule-1 (ICAM-1) and IL-8 expression in epithelial cells [67]. Amorphous SNP closely interacts with soluble serum proteins, as well as cellular membrane, resulting in more pronounced oxidative stress and pro-inflammatory effects in macrophages [68].

Here we also observe a dependence of the membrane-NPs interactions on the NPs size. Thus, although AuNP of 20 nm (AuNP20) could be internalized in cytosolic vacuoles, AuNP70 are restricted to the cell membrane. However, both particles induce apoptosis *via* caspase-dependent mechanisms. AuNPs induce degradation of the cytoskeletal proteins vimentin, lamin B1 and gelsolin [69].

Histidine rich glycoprotein is a widely distributed plasma alpha2-glycoprotein that interacts with ligands such as Zn^2+^, haem, tropomyosin, heparin, heparan sulfate, plasminogen, plasmin, fibrinogen, thrombospondin, IgG, FcgammaR and complement protein 1q (C1q), thereby modulating essential biological functions including the regulation of cell adhesion, migration, fibrinolysis, coagulation, complement activation, clearance of the immune complexes, as well as clearance of the apoptotic cells [70]. Histidine rich glycoprotein is a modular protein consisting of an N-terminal cystatin-like domain (N1 N2), a central histidine-rich region flanked by proline-rich sequences, and a C-terminal domain. The N1 N2 domain binds to cell-surface heparan sulfate. The histidine-rich region facilitates the cell-surface binding capacity by interacting with Zn^2+^ [71]. This glycoprotein is found in excessive amounts in human plasma and constitutes the most abundant element of the SiO_2_-NP hard corona. The depletion of histidine rich glycoprotein and kininogen-1 from human plasma or their exhaustion in plasma by the increase of NP concentrations lead to a heterogeneous hard corona which is mostly formed by fibrinogen, high density lipoproteins, LDLs, IgGs and kallikrein, which ultimately allows the binding of NPs to macrophages [72]. This is another example of a constant and dynamic interaction between the NPs and their microenvironment with immediate effects on NPs biological actions.

Well known heterodimeric membrane glycoproteins, integrins, consist of non-covalently associated alpha and beta subunits. These contact points modulate the attachment of the cells and their migration on the surrounding ECM. Integrins also regulate the cell to cell interactions under both physiological and pathological conditions affecting key biological functions such as proliferation, differentiation and apoptosis [73]. Ultrafine particle-induced proliferation is modulated by the activation of beta1-integrins that is mediated also through the EGFR signaling. The redirection of the cell into apoptotic or proliferative pathways is triggered *via* the modulation of interaction of EGFR and Bcl-2. Downstream of EGFR, the activation of MAPK ERK 1/2 promotes proliferation in a manner dependent on beta1-integrin, whereas on the other hand, the phosphorylation of JNK 1 and 2 is associated with the induction of apoptosis [74]. NP-induced proliferation can be mediated by PI3K and Akt. Moreover, overexpression of a variant type of Akt, and pretreatment with an Akt inhibitor, reduces NP-specific ERK1/2 phosphorylation. Thus, the resultant effect is the promotion of the NP-induced proliferation. The activation of a pathway by carbon NPs is triggered by ligand receptor binding or on cell adhesion to ECM proteins [75]. Akt plays an integral role in a wide range of intracellular signaling pathways that are mainly initiated by a variety of extracellular signals. As mentioned above, these signals are transferred *via* several classes of membrane-bound receptors [76]. Thus, when recognizing and interacting with cell membrane receptors, NPs can induce directly or indirectly activate intracellular pathways, *via* ECM proteins or kinases [77]. Additionally, exosomes, extracellularly secreted membrane vesicles that measure 30 to 100 nm in diameter, function in intercellular signaling by transporting different membrane and cytosolic molecules, including hyaluronan and its synthesis machinery [78]. They are also involved in transferring genetic materials, and modulation of immune response. Evidence indicates that they can be used as not only therapeutic agents targeted against disease but also diagnostic biomarkers for pathologic conditions [79]. Actually, NP-induced exosomes are signaling mediators, which provoke the activation of T helper cell type 1 and inflammatory responses [80].

### Macrophage recruitment

Macrophages are a type of immune cells that engulf and digest cellular debris, foreign substances, microbes, as well as cancer cells. Thus, they are also the “first responders” to NPs [81]. NP uptake is enhanced in M2-polarized primary human monocyte-derived macrophages compared to the M1 cells. M2 polarization promotes the NP uptake in the monocytic THP-1 cell line [82]. Rapid recruitment of macrophages is triggered by the activation of the complement system within injured tissues, such as in wounds and burns, by antigen/antibody (Ag/Ab) interaction. Such activation results in generation of chemotactic complement cleavage peptides, including C5a and C3a which induce extravasation of blood monocytes and maturation of these monocytes into macrophages [83]. Alpha-galactosidase is a glycoside hydrolase enzyme that is widely distributed in human. Alpha-galactosidase (Alpha-gal) NPs prepared from glycolipids with multiple alpha-gal epitopes, phospholipids, and cholesterol, cause extensive macrophage recruitment. Recruited macrophages reach Alpha-gal NPs as the result of interaction between the Fc “tails” of anti-Gal coating Alpha-gal NPs coated Alpha-gal NPs and FcgammaR on these macrophages. The binding of Alpha-gal NPs to macrophages *via* Fc/FcgammaR interaction activates macrophages. These NPs may further be incorporated into biodegradable scaffold materials such as natural or recombinant collagen sheets, dressings [83]. As anti-Gal ubiquitously present in humans, activation of the recruited macrophages by interaction with anti-Gal coated Alpha-gal NPs may induce angiogenesis, fibroblast migration, collagen deposition and effective recruitment of stem cells [84] (Fig. 4). Indeed, platelet-derived growth factor (PDGF)-AA, PDGF-BB, EGF, TGF-β1, monocyte chemoattractant protein-1 (MCP-1), interferon gamma (IFN-γ)-induced protein-10 (IP-10), IL-1α, IL-1β, and IL-15 are effective on both type I collagen and hyaluronan production in ECM remodeling. Some chemokines, such as MCP-1 (CCL2), regulates on activation, normal T cell expressed and secreted (RANTES; CCL5), eotaxin-2 (CCL24), IP-10 (CXCL10), or fractalkine (CX3CL1) significantly induce the type I collagen or hyaluronan production [85]. Overexpression of the recently identified glycophosphatidylinositol-anchored protein CD109, a novel TGF-β co-receptor and inhibitor of TGF-β signaling, has been shown to reduce inflammation and improve collagen organization in wound granulation tissue [86]. Indeed, TGF-β is an indispensable cytokine in tissue fibrosis. Despite both direct wound-healing and antibacterial effects of AgNPs, it has been shown that AgNPs do not induce TGF-β production [87]. Furthermore, AgNPs are predominantly responsible for regulating deposition of collagen and they generate an excellent alignment in the healing process [88]. These particles are deposited mainly to the terminal bronchial/alveolar duct junction region of the lung where they interact with ECM and also with the epithelial cells, independent from the particle size. However, treatment with smaller particles, 20 nm AgNPs, results in an elevated silver burden in bronchoalveolar lavage fluid macrophages [89]. The citrate-coated VSOP binds strongly to the cell surface, as well as to the apoptotic membrane vesicles in a manner dependent on hyaluronan expression. Since, hyaluronan synthesis is largely dependent on glucose levels, in a microenvironment where glucose is low, negatively charged hyaluronan synthesis decreases, attenuating the VSOP attachment to THP-1-derived macrophages [47].

Increased fibroblast proliferation and collagen synthesis are the cardinal signs of fibrosis. PDGF, synthesized by the macrophages is a potent mitogen and acts as chemoattractant for fibroblasts. The extent of the responsiveness of fibroblasts to PDGF is modulated by the proportion of cell surface PDGF receptor α (PDGF-Rα) number relative to the PDGF-Rβ number. The PDGF-Rα is upregulated by IL-1β which stimulates the expression of PDGF-AA, PDGF-AB, and PDGF-BB isoforms and ultimately promotes the lung fibroblast proliferation. Even though, both IL-1β and TGF-β1 are synthesized *via* the particle-stimulated macrophages, TGF-β1 has opposing effects. The macrophages treated with TiO_2_, chrysotile asbestos, or residual oil fly ash have 3-, 6- and 20-fold enhanced binding capacity to PDGF-AA, respectively. These NP-activated macrophages initiate fibrotic response by increasing fibroblast PDGF-Rα expression *via* IL-1β [90]. The remodeling of ECM is largely mediated by fibroblasts as the major cell type controlling ECM network. Innate cytokine responses may be critical in non-allergic/non-autoimmune disease, and they enable environmental agent exposure mechanisms that are independent of adaptive immunity [91]. Pulmonary alveolar macrophages may participate in the pathogenesis of acute inflammatory lung injury by the secretion of monocyte chemoattractants including MCP-1 [92]. Monocyte chemotactic protein-1-induced protein 1 (MCPIP1) plays a critical role in fibrosis induced by SiO_2_. MCPIP1 promotes the autophagic process in macrophages in response to silica exposure. Enhancement of macrophage autophagy further increases the pro-fibrogenic stimulatory effect. Eventually, macrophages act as paracrine effectors to modulate fibroblast proliferation and migration and macrophage autophagy plays a central role in these effects [93]. Accumulation of carbon nanomaterials in macrophage lysosomes, leading to lysosome membrane destabilization, indicates reduced autophagic degradation. This process is suggested to be a potential mechanism of the toxic effects of nanomaterials on cells [94].

In summary, NPs can recruit macrophages, activate them and are taken up by these immune cells. In some cases, autophagy of macrophages can be enhanced. *Via* activation of macrophages and subsequent fibroblast proliferation, NPs can indirectly re-model the ECM. These data indicate that a feedback mechanism involving macrophage and fibroblast responses which are mediated through ECM-dependent signaling determines NPs’ toxic effects in some tissue types.

### The role of extracellular matrix metalloproteinases in nanoparticle induced immune response

ECM matrix metalloproteinases (MMPs) are a family of zinc-dependent neutral endopeptidases. They are designated to degrade all matrix components, and are thereby involved in both physiological and pathological conditions [95]. Thus, MMPs act as extracellular proteases that modulate, for example, the immune response in pulmonary inflammatory processes. In the case of *Cryptococcus* infection the MMP-12 levels are strongly correlated with the expression of the macrophage- and neutrophil-attracting chemokine CCL2 mRNA [96]. It is noteworthy that TiO_2_ NPs induce the expression of elastase-induced MMP-12 mRNA in the lung [97] and in murine macrophages [98]. Carbon black (CB) NPs induce perivascular or peribronchial infiltration, increase inflammation and expression of the oxidative stress marker heme oxygenase-1 (HO-1) mRNA in ECM and increase the protease MMP-12 mRNA, as well as protein expression in alveolar macrophages. These particles also aggravate elastase-induced perivascular or peribronchial inflammation [97]. However, TiO_2_ and CB NPs elicit distinct apoptotic pathways [99] and oxidative stress, that can be determined by the elevation of the HO-1 expression and MMP-12 stimulation following the CB NPs exposure, but not after the TiO_2_ NPs treatment [100]. After incubation with 7 nm ultrafine TiO_2_ particles for 1 day, the expression of MMP-9 and MMP-10 genes and cell-matrix adhesion molecules, fibronectin-1, integrin subunit beta-6, and mucin-4 are altered in keratinocytes [101]. Furthermore, both AgNPs with a diameter of 20 nm (AgNP20) and AgNPs with a diameter of 70 nm (AgNP70) inhibit *de novo* protein synthesis. Both forms of AgNPs do not significantly affect ROS production, but AgNP20 significantly increases the CXCL8 chemokine (IL-8) generation. In addition, AgNP20, but not AgNP70, induce the release of albumin and MMP-9/gelatinase B [102]. When treated with AgNP20, the size of the neutrophils increase and transmission electronic microscopy results indicate that within 1 day of exposure, AgNP20 can rapidly interact with the cell membrane, penetrate into neutrophils, localize in vacuole-like structures, and are randomly distributed in the cytosol [103]. The cellular MMPs and tissue inhibitors of matrix proteases (TIMPs) strictly regulate the integrity of generated matrix at three distinct stages of elastic matrix synthesis [104]. Thus, TIMP-1 as an endogenous inhibitor of MMPs is capable of regulating the cleavage of ECM components, as well as membrane and secreted proteins. MMP-1 is an important factor in matrix homeostasis and involved in TiO_2_ NP associated effects. Thus, TiO_2_ NPs potentially stimulate and also modulate the MMP-1 expression and activity, partly through an IL-1β-dependent pathway [105]. Indeed, during inflammation and connective tissue destruction, IL-1β acts as a central mediator and also activates articular chondrocytes resulting in the release of MMP-1. In some diseases this involves connective tissue deregulation as IL-1β activated MMP-1 dismantles the collagen scaffold [106].

Fibrosis develops as the result of the abnormal accumulation of the ECM and ineffective clearance of fibroplasia. CD4+CD25+Foxp3+ regulatory T cells (Tregs) are immunosuppressive lymphocytes that are highly expressed in the fibrotic tissues. The expression of MMPs and TIMPs is altered depending on the depletion of Tregs; MMP9 and TIMP1 are decreased, whereas MMP2 and MMP14 are increased. Nevertheless, the MMP9/TIMP1, MMP13/TIMP1, and MMP14/TIMP2 are markedly elevated in association with resolution of fibrosis [107]. Survival of the Treg subset is dependent on the cytokines IL-2 and TGF-β whose syntheses are impaired in autoimmunity. In the pro-inflammatory microenvironment, the conversion of Tregs into effector cells is enhanced. Inert biodegradable NPs loaded with TGF-β and IL-2 and targeted to CD4+ cells can induce CD4+ Tregs conversion, *in vitro* and expand the CD4+ Treg population, *in vivo* [108]. However, the survival rate of induced Tregs with cytokine-loaded NPs are prolonged leading to retention of their suppressive phenotype even in the presence of proinflammatory mediators [108]. In addition to the effects on Tregs, the excessive bioavailability of TGF-β promotes elastic matrix synthesis. Furthermore, TGF-β1 and hyaluronan oligomers together induce much greater assembly of mature elastin fibers when provided concurrently [109]. Cerium oxide (CeO_2_) NPs also interact with the MMPs regulation. These NPs, introduced to lung epithelium through diesel exhaust exposure, significantly increase fibrotic cytokine TGF-β1 and osteopontin production by alveolar macrophages. Upon exposure to NPs, the collagen degradation enzymes, MMP-2 and -9 and the TIMP markedly increase. Moreover, CeO_2_ induces increment in phospholipids in bronchial alveolar lavage fluid and elevates hydroxyproline content associated with collagen fiber deposition in lung tissue in a dose- and time-dependent manner. Eventually MMP-2, MMP-9 and MMP-10 expressions increase in fibrotic regions [110]. Although, amorphous SiO_2_ (aSiO_2_) and CeO_2_ particles induce a dose-related inflammation, cytotoxicity, inflammatory cytokines, MMP-9, and tissue inhibitor of MMP, aSiO_2_ coating significantly reduces CeO_2_-induced inflammatory responses and attenuate phospholipidosis and fibrosis [111]. So far, experiments regarding the NPs, MMPs, TIMPs and immune response show an irregular picture because many different players interact with each other. Mainly, for metal-based NPs it has been shown that they increase chemokine concentration which in turn can influence sub-groups of MMPs which then may remodel the ECM in one or the other way depending on the specific MMP sub-group and the presence of different chemokines or cytokines. In summary, NPs-dependent signaling may seriously impair the ratio between MMPs and TIMPs which results in pathological turn-over of the ECM and facilitation of inflammatory processes.

### Lysyl oxidase crosslinking of elastin matrix and nanoparticles

**Fig. 5:**
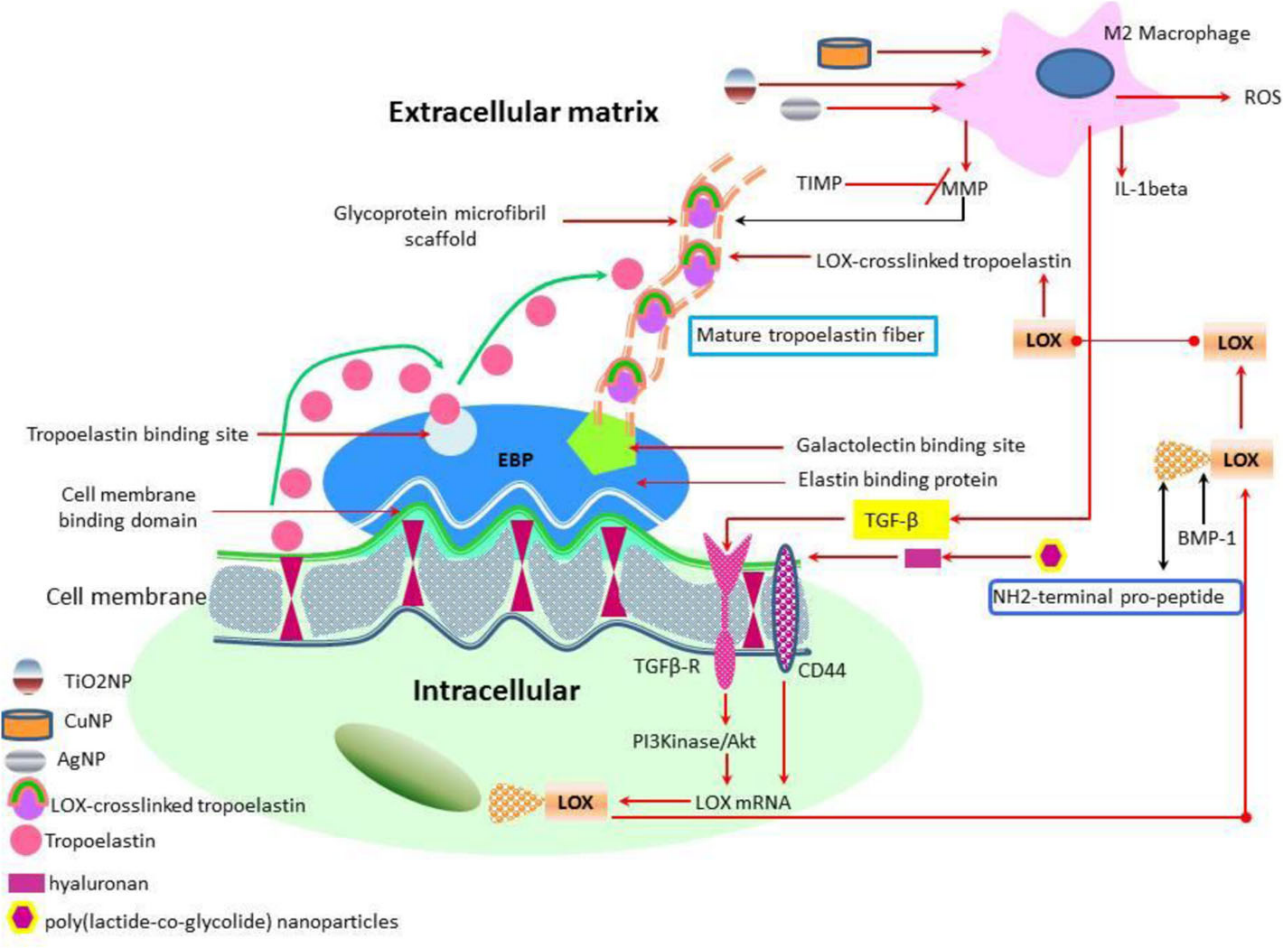
Effect of metal NPs and hyaluronan oligomers on the elastic fiber formation in the ECM. Initially, the tropoelastin molecule is secreted from the cell and binds to the elastin binding protein (EBP) on the cell membrane. Tropoelastin deposition and crosslinking by LOX on the glycoprotein microfibrils form mature tropoelastin fibers. (*ROS: reactive oxygen species, MMP: matrix metalloproteinase, TIMP: tissue inhibitors of matrix proteases, IL-1beta: interleukin-1beta, TGF-beta: transforming growth factor beta, BMP-1: Bone morphogenetic protein-1/Tolloid metalloproteinase, PI3Kinase: Phosphoinositide 3-kinase, LOX: Lysyl oxidase)*

Elastin, a fibrillar protein, is a key component of the ECM surrounding smooth muscle. The formation of the mature elastin fiber in the ECM demands the organization of its soluble precursor molecules, designated as tropoelastin, into a polymer characterized by a high level of crosslinking. The tropoelastin molecules however, do not have the innate ability to self-assemble but need helper proteins to correctly assort their multiple crosslinking sites to facilitate this process. Thus, versican, a large ECM proteoglycan in collaboration with hyaluronic acid, plays a key role in elastin precursor recruitment and fiber assembly. The precise interaction of this helper protein with tropoelastin molecules is favoured by its highly anionic nature enabling it to bind to oppositely charged regions of tropoelastins [112]. Indeed, the tropoelastin molecules are assembled from mostly hydrophobic amino acids, with lysine containing hydrophilic domains. The hydrophilic lysine residues play an important role in the crosslinking of tropoelastin molecules, which is a critical step of the elastic matrix assembly process [113]. In this context, lysyl oxidase (LOX), a copper-dependent amine oxidase plays an important role in the stability of the elastic matrix by catalyzing the crosslinking of tropoelastin into mature elastic fibers [114]. NPs cause a significant increase in LOX production [115]. The increase in LOX production is consistent with desmosine crosslinking of elastin [116]. Thus, increased crosslinking of elastin precursors is responsible for the increased deposition of elastic matrix. Interestingly, PLGA NPs encapsulated with varying percent loadings of hyaluronan oligomers induce dose dependent increase in elastic matrix synthesis. Furthermore, these NPs enhance LOX production, and lead to improved deposition of a microfibrillar network [115]. Elastin matrix deposition stimulation by Cu^2+^ ions and enhanced crosslinking *via* stimulation of LOX simultaneously occur in hyaluronan and copper NP-treated elastin matrix [109] (Fig. 5). Therefore, the targeting of elastin matrix by specific NPs can improve its stability that is crucial in human disease as is the case of vascular inflammation [112].

### Interaction of nanoparticles with biological barriers

During the ontogeny of cavity containing tissues, an ECM-dependent mechanism orients and polarizes the cells in the tissue, in order to obtain phenotypes that support physiological functions. The epithelium and its’ specialized subtype, the endothelium, are lining the body’s cavities and blood vessels, including the blood-brain barrier. They represent the main trafficking avenues studied in nanomedicine. Furthermore, NPs were characterized in terms of dimensional analysis, polydispersity and zeta potential, morphology, encapsulation efficacy, and loading capacity [117]. It is noteworthy that basal membranes, the highly specialized ECM structures, are crucial components of all biological barriers [2, 4, 10].

The epithelium represents a monolayer comprised of cells that delineate a cavity or even a surface. In this type of tissue, the cells have a distinct 3D orientation. They have an apical type of plasma membrane that is fronting the lumen or the surface, a plasma membrane that has a lateral position touching the surrounding cells, and a plasma membrane at the base, contacting the basal membrane, constituting highly specialized form of ECM. This specific orientation is synchronized between the cells that form the tissue, namely the cavity where the apical plasma membranes of neighboring cells are radially polarized. The main mechanisms for cell polarization are sustained by the interaction of cells with ECM and with the surrounding cells [118].

**Fig. 6:**
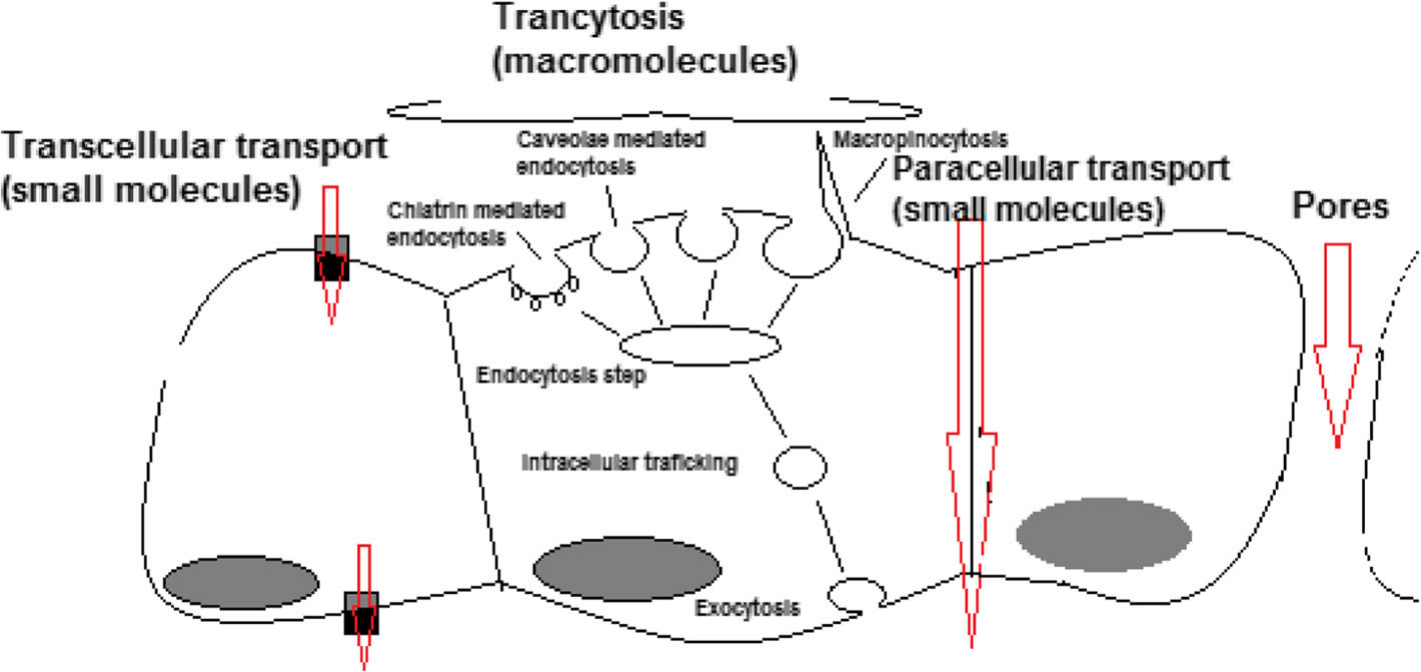
Transport mechanisms within tissue. Small molecules can be transported either transcellularly or paracellularly, while macromolecules and NPs use transcytosis [121]

Recently, it was shown that defects in ECM signaling induce an inverted polarity between apical surfaces and surfaces facing the ECM. Thus, in a Madin Darby Canine Kidney (MDCK) epithelial cellular *in vitro* model, it was reported that there is a molecular switch that actually controls the polarity orientation. At the ECM/cell interface signals are generated which involve a b1-integrin/FAK/p190RhoGAP complex that down-regulates the RhoA/Rho-associated protein kinase/Ezrin (RhoA/ROCK/Ezrin) pathway [119]. In this manner, podocalyxin, a protein important for cell polarity orientation [120] is removed from the cell surface adjacent to ECM and transcytosed to the apical membrane where there is the initiation site for lumen formation. Transcytosis occurs when membrane-bound carriers transport molecules in a selective manner between one part of the cell to another. Inhibition of this mechanism retains podocalyxin at the ECM interface and disturbs polarization and motility. Collective orientation and epithelial polarization are controlled by ECM-derived signals [119]. Importantly, this mechanism may be utilized for NP transport. The mechanics of transport is seminal for several systems inducing, epithelial-based tissue and hepatocyte barrier. Small molecules can be transported either transcellularly or paracellularly, while macromolecules and NPs use transcytosis (Fig. 6).

Transcytosis is used by the epithelial cells in the immune processes, in nutrient absorption, and plasma membrane biogenesis. Endothelium and cells of the endocrine system also rely on transcytosis processes [121].

#### Endothelium barrier

**Fig. 7:**
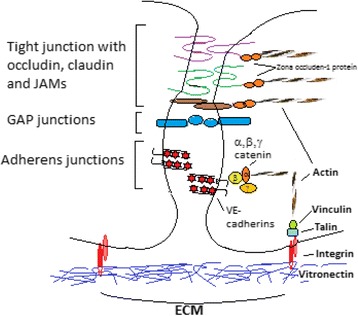
Endothelial cell interaction with ECM. Barrier function is maintained by endothelial cells inter-cellular interactions and adherence to ECM. Occludin, claudins, and junctional adhesion molecules (JAMs) comprise the tight junctions, while VE-cadherin interactions stabilize adherens junctions. Connexins develop gap junctions. Junctional stability is intracellularly maintained by linking with the actin cytoskeleton intermediated by catenins (e,g, β-catenin, α-catenin; γ-catenin;) or zona occluden-1 protein. Endothelial cells interact with ECM matrix protein (fibronectin or vitronectin) through integrin receptors. Proteins like talin and vinculin link integrins’ cytosolic domains with actin cytoskeleton, these proteins are also involved in integrin-mediated signaling

The endothelium barrier, consisting of microvascular endothelial cells at blood-vessel interface, controls the essential functions of body organs, regulating the volume of tissue fluids and supply of nutrients for homeostasis. Indeed, the endothelial cell may be characterized as a cellular antenna that is sensitive to signals/factors generated in the blood, to the basal membrane, to the sub-endothelium, as well as to the interacting cells. Endothelial cells are distinct from epithelial cells due to their semipermeable characteristic. This characteristic is necessary for the continuous controlled paracellular and transcellular pathways that need a transendothelial protein gradient (colloid osmotic gradient). At this level, interendothelial junctions (IEJ) have proteins that pair to specific ECM components and have the role of limiting the transport of plasma proteins by paracellular pathways. Endothelial cells have an abundant expression of integrins that are actually receptors for subendothelial ECM proteins. At the sites where integrins link to ECM, focal contacts or focal adhesions sites are generated. The focal contacts in collaboration with IEJ induce cell shape alterations and control paracellular permeability (Fig. 7). Indeed, the endothelial cells continuously remodel ECM in mature vessels by secreting ECM constituents which stimulate angiogenesis and vasculogenesis [122]. When the ECM interaction with integrins is established, endothelial cells do not proliferate and/or migrate and become quiescent, which is an essential characteristic for the endothelial barrier [123].

Beside other mechanisms, the diameter of the NP is an important attribute to enable NP to overcome the various *in vivo* barriers including ECM components [124]. In the wide framework of nanomedicine, the mechanism types of endothelial transcytosis, similar to epithelium transport, depend on NPs’ functionalization, size and charge. Thus, endocytic pathway is size-dependent. It is indicative that endothelial cells internalize 20 nm NPs by caveolae-mediated endocytosis, whereas the 40 nm NPs are uptaken through clathrin-mediated internalization and micropinocytosis [125]. Importantly, the sub-endothelial ECM contains molecules that regulate transportation. An illustrating example is the specific activity of polymorphonuclear neutrophil-derived (PMN-derived) and monocyte-derived myeloperoxidase (MPO) which catalyze nitrotyrosine formation, a characteristic trait of vascular inflammation. The MPO molecule is transcytosed and is subsequently concentrated to the subendothelial ECM of vascular tissues. The MPO locates to the ECM then specifically catalyzes the nitration of discrete ECM protein tyrosine residues; the main target protein in this case being fibronectin. Interestingly both unfractionated heparin and its low-molecular weight derivative, enoxaparin, in standard endothelial cells and in rat aortic tissues significantly inhibit MPO binding and protein nitrotyrosine formation. Baldus et al., therefore suggest that “Endothelial transcytosis of MPO confers specificity to vascular ECM proteins as targets of tyrosine nitration” [126]. Another important research topic regarding the endothelium barrier is the study of interchange among migrating leukocytes, ECM components and NPs during inflammation. Thus, upon activation of human neutrophils the resulting generation of NO converts intracellular glutathione (GSH) to S-nitrosoglutathione (GSNO), a biological pool for controlled NO release [127]. Alternatively, NO induced oxidation of ECM components, including fibronectin stimulates neutrophil adhesion to ECM decreasing their migratory abilities [128]. Therefore, a targeted uptake of GSNO to immune cell’s intracellular compartments will stimulate immune response without deleterious side effects to ECM. Nanoformulated GSNO with Eudragit as a carrier was tested on THP-1 human monocytic cell line with beneficial results. The mechanisms for transportation was identified as clathrin- and caveolae-mediated endocytosis [129].

#### Hepatocyte barrier

Hepatocytes, like other epithelia, are organized to perform and control the exchange of molecules between two distinct compartments; hepatic parenchyma and capillary system [130]. Due to the central role of the hepatic parenchyma in the metabolism of xenobiotics, many studies have examined the issue of NP uptake and interaction with the liver tissue [131]. Total hepatic collagen content is specifically reduced by the cationic lipid NPs loaded with small interfering RNA to the procollagen alpha1 gene without detectable side effects. Therefore, this application may be a potentially qualifying therapy for fibrotic liver diseases [132].

**Fig. 8:**
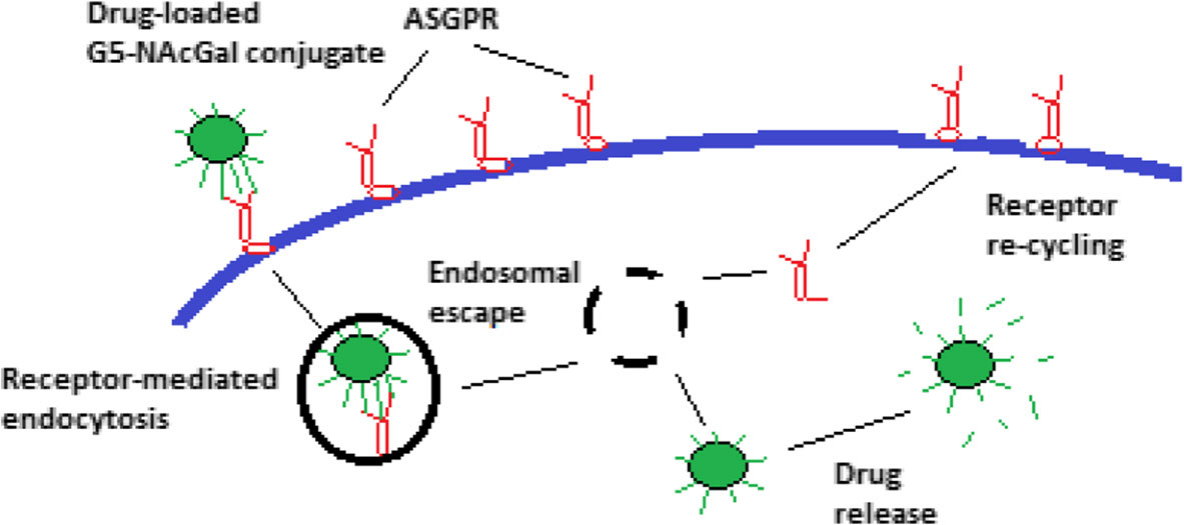
Drug-loaded G5-N-Acetylgalactosamine (NAcGal) conjugate that can bind to the asialoglycoprotein receptor (ASGPR) expressed on the surface of hepatic cancer cells. Binding of the drug-loaded conjugate to ASGPR triggers receptor-mediated endocytosis followed by endosomal escape and release of the drug, ASPGR recycles to be again expressed on the cell’s surface

In the same *in vitro* experimental model HepG2, PAMAM-NH_2_ dendrimers were used as N-Acetylgalactosamine (NAcGal)-targeted carriers for chemotherapeutic agents. This approach demonstrated that the uptake of dendrimers in HepG2 cells occurs *via* asialoglycoprotein receptor (ASGPR)-mediated endocytosis [133] (Fig. 8). The above mentioned interactions of NPs with biological barriers and specifically their basal membrane component, exhibit extraordinary complexity and highlight the necessity for further study.

## Conclusions

The focus of the scientific community on the use of nanotechnology for technical and consumer applications and in medical diagnostic and treatment during the last decades has resulted in establishing a substantial database. We need to further deepen our knowledge on the interaction of various NPs with specific biological barriers and compartments in optimizing internalization and site specific drug release. However, although it is clear that the content of the extracellular microenvironment critically affects the behavior of NP and their interaction with biological barriers, there is still limited knowledge about the mechanisms and role of different biomolecules in mediating the interaction of these materials with the assembly of ECM. ECM is a highly organized complex network of structural proteins, proteoglycans and glycosaminoglycans. Alteration in the expression of ECM molecules can ultimately affect the immune cell activation, differentiation and survival. These modifications eventually influence the propagation of the inflammatory response. Depending on the targeted tissue, the NPs encapsulated drugs aim to cross specific barriers, including endothelium, or hepatocyte barrier and, at each of these points, there are discrete interactions of the NPs with ECM components. Thus, functionalization and polarization of the drug carrier NPs with suitable peptides and other compounds concurrently enhances their therapeutic effects, by facilitating the transport and bioavailability of encapsulated agents through the ECM, as well as cellular compartments. Taking into account the continuously growing release of various NPs into the environment there is a demanding necessity to define and categorize ECM/NPs interactions and to examine their relevance regarding toxicity and inflammation in various biological processes. Therefore, future research should be focused on elucidating the molecular transport mechanisms that will provide groundwork for coherent design of more effective nano-carriers, improve their performance and target specific intracellular compartments.

## Abbreviations

Ag/Ab: Antigen/antibody
AgNPs: Silver NPs
Akt: Protein kinase B
AMPK: Adenosine monophosphate kinase-related kinase
anti-Gal: Anti-Galactose alpha 1,3-galactose
ASGPR: Asialoglycoprotein receptor
aSNP: Amorphous silica NP
ATF-6: Activating transcription factor-6
AuNP: Gold NPs
C1q: Complement protein 1q
CB: Carbon black
CCL24: Eotaxin-2
CeO_2_: Cerium oxide
CX3CL1: Fractalkine
ECM: Extracellular matrix
EGFR: Epidermal growth factor receptor
ERK: Extracellular signal regulated kinases
FDA: Food and drug administration
G-CSF: Granulocyte colony-stimulating factor
GM-CSF: Granulocyte-macrophage colony stimulating factor
GSH: Glutathione
GSNO: S-nitrosoglutathione
GSR1: Glutamine sensing regulator 1
GSTM3: Glutathione S-transferase Mu 3
HO-1: Heme oxygenase-1
ICAM-1: Intercellular adhesion molecule-1
Ig: Immunoglobulin
IL: Interleukin
IP-10: Interferon (IFN)-γ -induced protein-10
IRE1: Inositol-requiring protein-1
IRF5: Interferon regulatory factor 5
IκB: Inhibitor of kappa B
JNK: c-Jun NH2-terminal kinases
LKB1: Liver kinase B1
LOX: Lysyl oxidase
MAPK: Mitogen-activated protein kinases
MCP-1: Monocyte chemoattractant protein-1
MDCK: Madin Darby Canine Kidney
MFAP4: Microfibril-associated protein
MLR: Membrane/lipid rafts
MMP-1: Matrix metalloproteinase-1
MMPs: Matrix metalloproteinases
mtDNA: Mitochondrial DNA
N1N2: N-Terminal cystatin-like domain
NADPH: Nicotinamide adenine dinucleotide phosphate
NMSNs: Amino-functionalized mesoporous silica nanoparticles
NO: Nitric oxide
NOX: NADPH-oxidase
NP: Nanoparticle
ODN: Oligodeoxynucleotides
PAI-1: Plasminogen activator inhibitor-1
PDGF: Platelet-derived growth factor
PDGF-Rα: PDGF receptor α
PGC-1α: Peroxisome proliferator activated receptor gamma co-activator-1alpha
P-gp: P-glycoprotein
PI3K: Phosphoinositide 3-kinases
PLGA: Poly-(lactic-co-glycolic) acid
PMN-derived: Polymorphonuclear neutrophil-derived
POR: Cytochrome P450 oxidoreductase
RANTES; CCL5: Regulated on activation, normal T cell expressed and secreted
ROS: Reactive oxygen species
S1P: Sphingosine 1-phosphate
SP-D: Surfactant protein-D
Src: Steroid-receptor coactivator
TGF-β: Transforming growth factor-beta
TIMPs: Tissue inhibitors of matrix proteases
TiO_2_: Titanium dioxide
Tregs: Regulatory T cells
VEGF-A: Vascular endothelial growth factor-A
VSOP: Very small superparamagnetic iron oxide particles
Alpha-Gal: Alpha-galactosidase
